# An Umbrella Review of Body Image Concerns, Disordered Eating, and Eating Disorders in Elite Athletes

**DOI:** 10.3390/jcm13144171

**Published:** 2024-07-16

**Authors:** Scott J. Fatt, Emma George, Phillipa Hay, Nikki Jeacocke, Emily Gotkiewicz, Deborah Mitchison

**Affiliations:** 1 Translational Health Research Institute, School of Medicine, Western Sydney University, Campbelltown 2560, Australia; e.george@westernsydney.edu.au (E.G.); p.hay@westernsydney.edu.au (P.H.); deborah.mitchison@uts.edu.au (D.M.); 2 School of Health Sciences, Western Sydney University, Campbelltown 2560, Australia; 3 Mental Health Services, South Western Sydney Local Health District, Camden and Campbelltown Hospital, Campbelltown 2560, Australia; 4 AIS Performance, Australian Sports Commission, Bruce 2617, Australia; nikki.jeacocke@ausport.gov.au; 5 Department of Psychology, University of Pittsburgh, Pittsburgh, PA 15260, USA; emg196@pitt.edu; 6 Discipline of Clinical Psychology, Graduate School of Health, University of Technology Sydney, Ultimo 2008, Australia

**Keywords:** body image, disordered eating, eating disorder, athletes, sport

## Abstract

**Background/Objectives:** Several systematic reviews and meta-analyses have been conducted to date indicating a high prevalence of disordered eating in elite athletes and associated risk factors. However, the substantial time burden associated with locating and comparing these reviews, which are varied in methodology and sampling focus, may be a barrier for informing policy and best practice as well as directing future research. This umbrella review aimed to provide a summary of evidence across published reviews regarding the prevalence and risk factors for disordered eating (including body image concerns and eating disorders) in elite athletes. **Methods:** Five databases (CINAHL, PsycINFO, MEDLINE, Scopus, and SPORTDiscus) were systematically searched for peer-reviewed systematic reviews and meta-analyses that met the following inclusion criteria: (1) investigated prevalence and risk factors for disordered eating, (2) included a sample of elite athletes, and (3) available in English. The included studies underwent data extraction and risk of bias assessment using the AMSTAR 2. **Results:** The initial search identified 1828 articles that were screened for title and abstract and then full text, leaving 24 systematic reviews (including 10 meta-analyses). Disordered eating was prevalent across elite athletes, including males and females and across sport type. Elite athletes were at elevated risk for disordered eating and eating disorders but at lower risk for body image concerns versus non-athlete controls. Several risk factors were identified, including female gender, competing in lean sports, and experiencing career changes. Few reviews or meta-analyses examined perceived pressures within the sporting environment, and most had substantial risk of bias concerns. **Conclusions:** Elite athletes are at risk for the spectrum of disordered eating, and all should be considered for primary prevention and screening. Further research is needed regarding sport-specific versus general pressures and mediators of risk to better inform interventions.

## 1. Introduction

Disordered eating refers to disturbances in cognitions and behaviours associated with eating, exercise, and body weight, with associated distress and/or biopsychosocial impairment [1,2]. Often related to this are disturbances in perception, affect, and cognition directed towards one’s body (i.e., body image concerns [3]). Individuals who experience disturbances in these areas according to specific criteria with sufficient frequency and chronicity may meet diagnostic criteria for an eating disorder [4], whilst many others experience a broad spectrum of disordered eating and body image concerns without meeting diagnostic thresholds.

Athletes, like all individuals, may sit anywhere on this spectrum ranging from optimised eating, exercise, and body image through to disordered eating and clinical eating disorders [5]. In particular, elite athletes (i.e., national, international, professional, or NCAA D1 level [6]) may experience pressures regarding dietary control, exercise, and elevated body monitoring or awareness as part of their training and competition [7,8,9]. Additionally, the risks for potentially severe physical and mental health outcomes associated with disordered eating [10,11] may be elevated for elite athletes due to high energy expenditure and increased strain on their bodies during competition and training. This can lead to low energy availability (LEA) and relative energy deficiency in sport (REDs), with potentially severe impacts on an athlete’s health and performance [12,13]. It is unsurprising, therefore, that several studies to date have investigated prevalence and risk factors for the spectrum of disordered eating (including body image concerns and eating disorders) in elite athletes.

Findings to date have generally (and paradoxically) indicated elevated risk for disordered eating and eating disorders but also lower body dissatisfaction amongst elite athletes compared with non-athletes. Three meta-analyses were published at the turn of the century indicating that athletes (both elite and non-elite) were at elevated risk for disordered eating versus non-athletes [14]. This risk was greater for athletes participating in lean sports (i.e., sports that inherently promote a drive for leanness including aesthetic, endurance, anti-gravitational, and weight-class sports [15]) versus athletes participating in non-lean sports [16]. Interestingly, athletes (both elite and non-elite) have also tended to report significantly lower body dissatisfaction than non-athletes [16,17]. These findings were supported by later robust population-based case–control studies indicating elevated risk for eating disorders amongst elite athletes vs. non-athlete controls (13.5% vs. 4.6% [18]), with greater risk for those participating in lean sports versus non-lean sports [19]. Thus, meta-analytic and case-controlled findings indicate elevated risk for disordered eating prevalence amongst elite athletes.

There is a need for a better understanding of which athletes are at greatest risk and why to inform policy and best practise as well as interventions for prevention and treatment. Specifically, risk factors include biopsychosocial factors both internal and external to the athlete, which may precipitate disordered eating; moderating factors identifying which athletes may be more at risk and when; and mediating factors explaining how and why such factors lead to disordered eating [20]. It is unclear if the risk factors and interventions established in the general public will translate to elite athletes, given their unique experiences regarding eating, exercise, and body image [8]. In their overview of risk factors for disordered eating amongst elite athletes, Bratland-Sanda and Sundgot-Borgen (2013) [21] reported emerging evidence across quantitative and qualitative studies for links between general risk factors and disordered eating (genetics, age, pubertal status, body dissatisfaction, low self-esteem, personality traits, negative affect, eating disorders in the family, peer pressure, influence of media, bullying, physical or sexual abuse, drive for muscularity, anabolic-androgenic steroid use, and homosexuality). However, they also proposed *sport-specific* risk factors (weight cycling and dieting pressure, personality, early start of sport-specific training, traumatic events, coaching behaviour, and rules and regulations in sports) that may not be adequately addressed in current prevention and treatment programs. The clarification of the risk factors (including moderators and mediators) specifically relevant for elite athletes will guide the development of targeted and effective interventions.

A synthesis of current findings regarding prevalence and risk factors for disordered eating in elite athletes could help address this lack of clarity. Whilst Bratland-Sanda and Sundgot-Borgen (2013) [21] provided a useful overview, it lacked a systematic approach. In the last decade, further systematic reviews and meta-analyses have been published in this area [6,22,23]; however, they are varied in methodology and sampling focus (e.g., only male elite athletes, only athletes competing in lean sports, and only including studies published within a certain timeframe). There is a considerable time burden for policymakers, clinicians, and researchers associated with locating and synthesising these findings. Accordingly, an umbrella review (also called a review of reviews) can provide a rigorous summary of evidence for a specific research question by synthesising consistencies and contradictions across relevant systematic reviews and meta-analyses [24]. Such a summary of evidence of prevalence estimates and risk factors for disordered eating and body image concerns in elite athletes would help identify which athletes are most at risk and why, informing policy, best practice, and future research directions. Accordingly, this study aimed to review the existing systematic reviews (including meta-analyses) regarding the prevalence and risk factors for disordered eating (including eating disorders) and body image concerns across various populations of elite athletes.

## 2. Materials and Methods

The umbrella review was prospectively registered with PROSPERO (CRD42023442206) and has been reported in line with the PRISMA [25] guidelines as well as guidelines for conducting and reporting umbrella reviews [24]. https://www.crd.york.ac.uk/prospero/display_record.php?ID=CRD42023442206 (accessed on 12 July 2024). Two changes were made from the original registration. First, the review was planned to also compare reviews of interventions for preventing and treating disordered eating amongst elite athletes; however, due to a lack of intervention research meeting the search criteria (i.e., only one study across all of the included reviews and meta-analyses), the review was narrowed to only investigating prevalence and risk factors. Second, searches were originally planned and conducted across four databases (MEDLINE, Scopus, PsycINFO, and CINAHL); however, an additional sports-based journal database with which the authors were not originally familiar (SPORTDiscus), was found to be included in the search strategy of several of the systematic reviews we uncovered, and so, we decided to update our search strategy to include this database.

The inclusion criteria were (1) peer-reviewed reviews or meta-analyses that were written in English and described a systematic search and article selection process; (2) including and reporting on a sample of current or former elite athletes (national, international, professional, and NCAA D1); and (3) synthesised findings regarding prevalence or risk factors for disordered eating or body image concerns. Reviews that included athletes from various competition levels were included if additional syntheses or comparisons were conducted focusing only on elite athletes. Articles lacking a clear synthesis or comparison focusing on elite athletes, “grey literature”, and non-English articles were excluded. Following consultation with a health librarian, searches were originally completed on four electronic databases: CINAHL (EBSCO), PsycINFO (OVID), MEDLINE (OVID), and Scopus, including all years until the search date, 4 July 2023. An updated search of these databases and the addition of SPORTDiscus (EBSCO) was conducted on 9 January 2024. The population concept context method was used for the search strategy [26], including (1) index terms and (2) key term searches in the titles and abstract (see Supplementary S1 for the full search strategy). Population included terms relating to “athlete”, concept included terms relating to “disordered eating” or “body image”, and context included terms relating to “systematic review” or “meta-analysis”. The results were then limited to peer-reviewed articles written in English.

**Figure 1:**
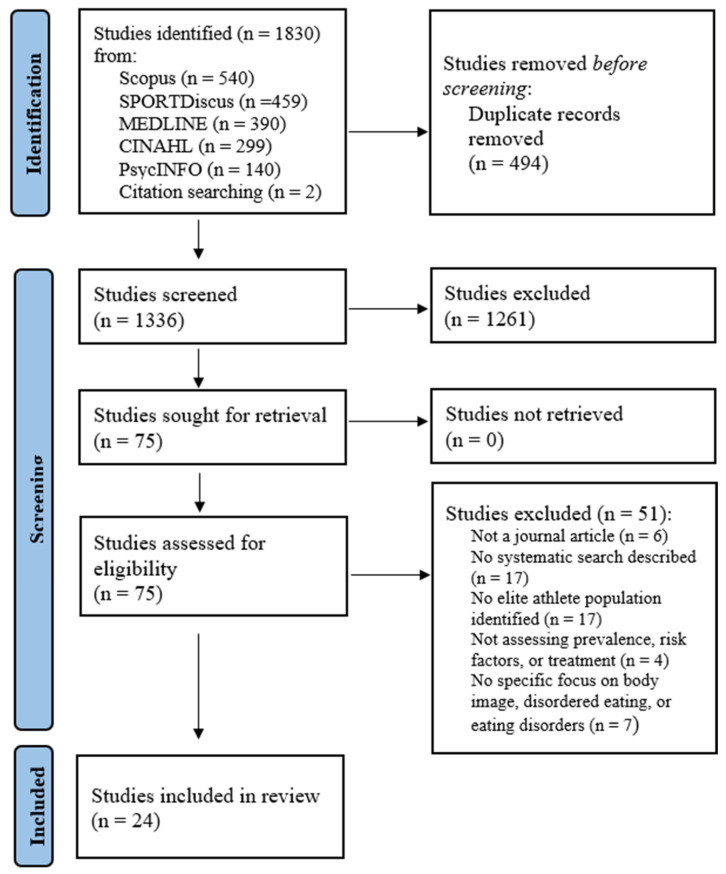
PRISMA flowchart for the search and screening process.

The search produced 1828 articles (1219 initially with an additional 609 from the updated search), which were imported into Covidence (Covidence, Melbourne, Australia) software, where 494 duplicate articles were automatically removed. Article screening was conducted independently at each level by S.F. and E.Go. using Covidence software. Following screening of each title and abstract, 73 full-text articles were assessed for eligibility against the inclusion criteria. Any discrepancies were addressed through discussion between the two reviewers and D.M. This left 22 articles, and following review of their reference lists, an additional two articles were identified, leaving a final total of 24 articles. See Figure 1 for PRISMA flowchart.

### 2.1. Data Extraction

Data were extracted for the 24 included articles by two authors, with S.F. extracting the data into a Microsoft excel sheet (Microsoft, Version 2406) and E.Go. checking the extracted data against the original articles. This included (1) citation details; (2) objective of the synthesis; (3) type of synthesis; (4) participant details and context; (5) inclusion criteria; (6) number of reviewers; (7) number of databases and search date; (8) publication date-range; (9) number of included studies; (10) instrument used for quality appraisal; and (11) outcomes (including prevalence, risk factors). Discrepancies were discussed among the two authors and, if needed, with an additional author, D.M.

The synthesis and presentation of outcomes can vary across umbrella reviews according to their research question(s) [24]. Findings are typically presented as a summary of evidence that highlights consistencies and contradictory findings across articles [24]. Given the aims of this review, a summary of evidence was synthesised for the (1) prevalence and relative risk of disordered eating and body image concerns across elite athlete populations as compared with non-athletes and (2) risk factors for disordered eating and body image concerns for elite athletes.

### 2.2. Risk of Bias Assessment

The included reviews and meta-analyses were assessed for risk of bias by two authors (S.F. and E.Go. or D.M.) using the AMSTAR 2 [27], which assesses risk of bias using 16 items across the critical domains of (1) pre-registration; (2) adequacy of literature search; (3) justification of excluded studies; (4) risk of bias in individual studies; (5) meta-analytic method; (6) risk of bias in interpreting the results; and (7) publication bias. Discrepancies were discussed among the authors and collectively agreed upon.

## 3. Results

The extracted data from the 24 included reviews and meta-analyses (published between 1999 and 2023) are presented in Table 1. Ten articles included meta-analyses. Most articles included athletes from a mix of sports (16/24), with some only including certain sports-type athletes such as dancers (three articles), cyclists (two articles), body builders (one article), jockeys (one article), and football players (one article). Two reviews only included studies of male athletes, three only included studies of female athletes, and nineteen included studies across genders. Nine reviews included only studies with elite athletes, and fifteen included studies with mixed levels of competition but included analyses focusing on elite athletes (e.g., moderating effect of competition level, sub-analyses for elite athletes, and large majority of elite athletes included).

### 3.1. Risk of Bias Assessment

There was 99.5% agreement between reviewers for the risk of bias assessment against the AMSTAR 2. Overall, most (21/24) of the included reviews and meta-analyses were at risk of bias for at least three of the critical domains (see Table 2 for a summary, with full details in Supplementary S2). The most commonly overlooked aspects, in order, were:(1) missing pre-registration of the review (21 articles—although this is a more recent practice); (2) inadequate consideration of risk of bias in interpreting the results (21 articles); (3) unclear justifications for the excluded studies (16 articles); and (4) inadequate or unclear search procedures (8 articles). Across the 10 meta-analyses, 8 did not account for risk of bias in their data-synthesis, and 5 did not appropriately assess for publication bias.

### 3.2. Overlap Analysis

Corrected covered area (CCA) analyses were conducted to assess the overlap of individual studies across the included reviews [46]. The CCA analyses indicated only slight overlap of individual studies for the reviews that investigated disordered eating (1.9%) and similar for those that investigated body image concerns (1.7%).

### 3.3. Prevalence of Disordered Eating (Including Eating Disorders) and Body Image Concerns

Several reviews included syntheses of the prevalence of disordered eating symptoms amongst elite athletes; however, none included prevalence of body image concerns. In a systematic scoping review of elite male athletes, prevalence for disordered eating ranged from 0–85.8% and clinical eating disorders from 1.3–32.5% [6], with Eating Disorders Not Otherwise Specified (EDNOS) the most common eating disorder diagnosis (up to 90%). Exercise addiction was also highly prevalent in a systematic review of male and female athletes, with primary exercise addiction ranging from 1 to 59% and secondary exercise addiction ranging from 1 to 80%, with no conclusive evidence of differences between elite and non-elite athletes [43]. Disordered eating was also common following retirement, with a prevalence of 42–65% in systematic review of retired elite athletes [37].

Syntheses of prevalence data for disordered eating were also available for specific sports. In a meta-analysis with eight studies investigating disordered eating in dancers (mostly elite), disordered eating ranged from 0 to 45.5% and prevalence of an eating disorder ranged from 8.0 to 50.0% [28]. In a later synthesis with 27 elite dancer samples (and six non-elite dancer samples), the pooled prevalence for an eating disorder using diagnostic interviewing against DSM-IV criteria was 12.0% (95% CI: 10.0–14.2), with 2.0% (95% CI: 0.9–4.3) for anorexia nervosa, 4.4% (95% CI: 3.2–6.2) for bulimia nervosa, and 9.5% (95% CI: 7.6–11.8) for EDNOS [31]. When using screening questionnaires to measure levels of disordered eating, 0–13.6% of males (2 studies) and 7.4–50.0% of females scored above the cut-off score for an eating disorder [31]. A systematic narrative review of current and former professional footballers found disordered eating prevalence ranging from 26 to 74.3% [42].

### 3.4. Relative Risk for Disordered Eating (Including Eating Disorders) and Body Image Concerns in Elite Athletes

Results of the articles comparing elite athletes with controls are included in Table 3 (disordered eating) and Table 4 (body dissatisfaction). Most results (8/11 included comparisons) indicated elevated disordered eating risk for elite athletes compared with non-athletes. Initial meta-analyses mostly included female athletes, finding small but significant effects for greater disordered eating amongst elite athletes versus non-athletes (comparisons for elite athletes only included females [14]; females only [16]). This was supported by a systematic narrative review by Rice et al. (2016) [34], although this only included four relevant studies. However, a recent meta-analysis by Chapa et al. (2022) [23] including studies of female athletes from 2001–2021 (i.e., since the earlier meta-analyses) found no significant differences between female athletes and non-athletes for disordered eating, drive for thinness, restricting, or binge eating, with no moderating effect of competition level (i.e., elite vs. non-elite). In fact, drive for thinness was significantly lower for female athletes participating in non-lean sports versus non-athletes [23]. For males, a meta-analysis indicated significantly greater scores on the Eating Attitudes Test for male athletes versus non-athletes but not for overall disordered eating symptoms [33], although this meta-analysis had concerns for risk of bias across pre-registration, article screening, and consideration of risk of bias in interpreting the results. A later systematic scoping review with a wider search found significantly higher disordered eating amongst male elite athletes versus non-athletes in 7 out of 11 included studies [6]. A recent systematic review including both males and females found significantly higher disordered eating amongst elite athletes versus non-athletes in five out of nine included studies, with no differences in the other four [39].

Several articles focused on disordered eating in specific athlete demographics. Meta-analytic reviews indicated that dancers were at elevated risk versus non-athletes for both disordered eating and eating disorder diagnosis [31] and that competitive body builders were at elevated risk for muscle dysmorphia versus non-body builder resistance trainers [35]. Further, a systematic review found that disordered eating was significantly higher in retired elite athletes compared with non-athlete controls [37]. Contrastingly, a systematic review of *young* (i.e., under 25 years) elite athletes found no conclusive evidence of elevated disordered eating versus non-athletes [30].

In contrast to disordered eating, all six of the included reviews reported lower *body image concerns* in athletes versus non-athletes. Meta-analyses found significantly lower body dissatisfaction for elite athletes than non-athlete controls with small to medium effects [16,17,22,23], even whilst reporting significantly higher drive for thinness [16]. Similarly, systematic reviews indicated significantly lower body image concerns in female collegiate athletes [29] and in male elite athletes [6] versus non-athletes.

### 3.5. Risk Factors for Disordered Eating and Body Image Concerns Amongst Elite Athletes

**Risk factors for disordered eating and body image concerns are presented in Table 5 and Table 6, respectively.** The row for each article includes any risk factors that were assessed and identified as significantly and consistently associated with disordered eating (Table 5) or body image concerns (Table 6), with an “=” indicating no consistent evidence of a significant relationship.

#### 3.5.1. General Risk Factors

**Demographic characteristics.** Demographic risk factors investigated across the reviews and syntheses included gender, age, and disability. Six articles investigated gender, typically as a moderator for disordered eating and body image concerns. An early meta-analysis with mixed competition levels found that the *effect sizes* reflecting greater disordered eating in athletes versus non-athletes were significantly larger for males than females [14]. This indicates that despite greater absolute risk among women athletes, there is a greater relative risk among men in elite sport. Two later systematic reviews found that disordered eating was significantly higher for female versus male elite athletes in both adults [34] and young athletes [30]. A systematic review of exercise addiction in athletes (mostly elite) found mixed findings comparing males and females, suggesting no consistent differences [43]. For body image concerns, an early meta-analysis found that the *effect size* indicating lower body image concerns for athletes of mixed competition level versus non-athletes did not differ between males and females [17]; however, a recent meta-analysis reported significantly higher body dissatisfaction and sport-specific body dissatisfaction for female versus male athletes of mixed competition level [44]. Thus, female athletes typically reported higher disordered eating and body dissatisfaction than male athletes, whilst male athletes may experience greater relative risk for disordered eating when compared with non-athlete males, than female athletes compared with non-athlete females.

Six articles investigated age as a risk factor for disordered eating and body image concerns. One systematic review found a negative association between age and disordered eating across a couple of studies with elite athletes [34]; however, age did not moderate relationships in meta-analyses of disordered eating or body dissatisfaction in female athletes (mostly elite [23]), nor exercise addiction [43] or body image concerns [17] in male and female athletes (mostly elite). Further, a systematic scoping review in male elite athletes found evidence of greater risk of disordered eating for adult athletes but not adolescent athletes vs. non-athletes [6]. This was congruent with mixed findings for disordered eating risk in young (i.e., under 25 years) elite athletes versus non-athletes, and there was no conclusive evidence of a moderating effect of age [30]. Thus, relative risk for disordered eating may be greater for adult versus adolescent athletes; however, these findings are mixed.

Only one article included examined risk of disordered eating or body image concerns for para-athletes [32]. Across the two included studies, para-athletes reported significantly less positive body image perceptions than non-para-Olympic athletes (small effect [32]).

**Psychological factors.** Psychological risk factors for disordered eating investigated across the articles included body dissatisfaction and negative affect. Although body image concerns and disordered eating were often examined separately, two systematic reviews investigated their relationship with each other, finding that higher body dissatisfaction was associated with disordered eating risk in elite athletes generally [39] and male elite athletes specifically [6]. Similarly, self-objectification was identified as a risk factor for elite athletes even into retirement [37]. Negative affect was also identified as a risk factor for disordered eating, finding positive relationships with depression in elite male athletes [6] and negative affect in elite athletes generally [39]. Thus, greater body dissatisfaction and negative affect may be psychological correlates of disordered eating in elite athletes.

#### 3.5.2. Sport-Specific Factors

**Sport type.** Sixteen articles investigated sport type as a risk factor for disordered eating and body image concerns in athletes. Eleven out of twelve reported higher disordered eating risk for athletes participating in lean sports versus non-lean sports, including larger effect sizes for aesthetic versus ball sports, relative to non-athletes (meta-analysis in female athletes [14]); larger effect sizes for lean versus non-lean sports, relative to non-athletes (meta-analysis in female elite athletes [16]; meta-analysis of drive for thinness in female athletes [23]); higher disordered eating scores for lean sports versus non-lean sports (systematic review of young elite athletes [30]; systematic reviews of elite athletes [34,38,39]; systematic review of elite male athletes [6]); greater risk for high energy expenditure sports (systematic review of cyclists [41]; systematic review of retired elite athletes [37]); and higher exercise addiction for weight-sensitive sports (systematic review of athletes [43]). Contrastingly, a meta-analysis by Chapman and Woodman (2016) [33] found no significant differences for disordered eating between male athletes participating in lean versus non-lean sports. Specific sports, however, were at elevated risk, with greater effect size for disordered eating amongst male wrestlers versus non-wrestler athletes, relative to non-athletes [33]. Similarly, a systematic review by Arcelus et al. (2014) [31] suggested that ballet dancers may be at elevated risk for eating disorders and disordered eating compared with other elite dancers (non-ballet). Overall, athletes participating in lean sports appeared to be at elevated risk for disordered eating versus non-lean sports.

Findings for sport type as a risk factor for body image concerns in athletes were mixed. An initial meta-analysis found that sport type did not moderate the relationship of lower body dissatisfaction in athletes versus non-athletes [17]. Similarly, a later meta-analysis only including body silhouette measures of body dissatisfaction found no difference between athletes (mostly elite) participating in aesthetic versus non-aesthetic sports [44]. A recent meta-analysis also found no significant difference for body image concerns comparing lean and non-lean athletes (mostly female), despite four of the seven included studies reporting greater concerns for lean athletes [22]. However, a recent meta-analysis found that sport type did moderate the relationship between athlete status and body dissatisfaction for female athletes such that the athletes participating in lean sports reported higher body dissatisfaction than non-lean sports, relative to non-athletes [23]. Thus, elite athletes may be at lower risk than non-athletes for body dissatisfaction, although this relationship is only clear for athletes participating in non-lean sports (and not those participating in lean sports).

**Pressures within sport.** Four reviews included pressures from the sporting environment as risk factors for disordered eating. Greater pressure from coaches and teammates to lose weight or pressure to perform well was associated with higher disordered eating in elite athletes [39] and in elite cyclists [45]. Further, a systematic review by Roberts et al. (2022) [41] identified a theme across qualitative findings focusing on power-to-weight ratios for performance as a risk factor for disordered eating amongst competitive cyclists. Disordered eating was also identified to increase due to different stages across the competitive season for elite cyclists [45] and jockeys [40].

**Sport-specific psychological factors.** Four articles included the internalisation of certain sport-specific beliefs as risk factors for disordered eating. Athletes who endorsed beliefs about a single “ideal” body for performance in their sport reported higher disordered eating, including the ideal “cyclist body” [41] and a general drive for leanness to improve performance [39,45]. A systematic review by Buckley et al. (2019) [37] suggested that internalisation of these ideals could continue into retirement and, when coupled with changes in body composition, could precipitate disordered eating [37].

**Career changes.** Several disruptions or changes in athletic career were investigated as risk factors for disordered eating, including injuries, career dissatisfaction, and retirement. Three systematic reviews indicated that higher disordered eating was associated with greater risk of injury in elite athletes, although few studies were included in each of these reviews (elite athletes [34]; dancers [36]; retired elite athletes [37]). Contrastingly, a systematic review found mixed evidence of associations between injury risk and disordered eating across two studies looking at professional football players [42]. Two systematic reviews found that greater career dissatisfaction was associated with higher disordered eating in elite male athletes [6] and retired elite athletes [37]. Finally, retirement was also identified as a risk factor for disordered eating in systematic reviews of retired elite athletes [37] and professional football players [42]. Overall, injuries, career dissatisfaction, and retirement may be associated with higher disordered eating, although the directions of these relationships are unclear.

### 3.6. Factors with Emerging Evidence

Table 7 presents additional potential risk factors that were only mentioned across one review.

## 4. Discussion

This umbrella review compared findings across 24 systematic reviews, including 10 meta-analyses, regarding the prevalence and risk factors for disordered eating problems (including body image concerns and eating disorders) in elite athletes. Overall, elite athletes were at elevated risk for disordered eating but lower risk for body dissatisfaction compared to non-athlete controls. There was consistent evidence that athletes who were female, participated in lean sports, and had experienced career changes were at elevated risk for disordered eating. However, this review identified gaps in our understanding of the role of sport-specific pressures or mediating factors for disordered eating in elite athletes.

The first aim was to compare and summarise the prevalence of disordered eating and body image concerns in elite athletes across syntheses including, where examined, the relative risk compared with the general population. When compared to the general population, most of the included reviews and meta-analyses found elevated risk for disordered eating and eating disorders in elite athletes, generally with small effect sizes. In contrast, elite athletes consistently reported lower body image concerns on average compared with non-athlete controls. These findings align with a population-based case-controlled study, with male and female elite Norwegian athletes meeting DSM-IV eating disorder diagnostic criteria across all sporting categories [18]. Further, eating disorder rates were more prevalent in both male and female elite athletes compared with non-athlete controls [18]. Together, these findings indicate that any athlete, regardless of gender or sport type, may be at risk for disordered eating [5].

The second aim of this umbrella review was to identify risk factors for disordered eating and body image concerns amongst elite athletes. Bratland-Sanda and Sundgot-Borgen (2013) [21] proposed a set of general risk factors and sport-specific risk factors for disordered eating in athletes. Several of these factors were included in at least two of the reviews or meta-analyses, including being female, higher body image concerns (including self-objectification, body dissatisfaction, and weight and shape concerns), negative affect, participating in lean sports, pressures within sport (pressure to lose weight, high performance climate, changes across the competitive season, and power-to-weight ratios), sport-specific psychological factors (internalisation of athletic ideals and drive for leanness for sport performance), and career changes (injuries, career dissatisfaction, and retirement). These risk factors also aligned with several key themes identified across the qualitative literature indicating that pressures within the sporting environment, certain sport-specific beliefs, and career changes may contribute to disordered eating in elite athletes [47,48,49].

### 4.1. Implications

The higher prevalence of disordered eating across elite athletes challenges a common misperception of eating disorders affecting only a certain narrow demographic (e.g., white, young, and female) and constituting a narrow set of symptoms (e.g., severe weight loss, self-induced vomiting, etc. [50]). Sporting organisations, training staff, and clinicians should proactively consider risk for disordered eating amongst their athletes even if the athletes do not fit certain stereotypes. We echo recommendations from a recent narrative review by the International Olympic Committee (IOC) for preventing REDs (including disordered eating) in athletes [51], highlighting the need for education and screening across all athletes to improve early identification and intervention.

The widespread prevalence estimates across reviews also highlights a need for improvements regarding best practise for screening and assessing disordered eating in elite athletes. Congruent with the observations of Bratland-Sanda and Sundgot-Borgen (2013) [21], the reviews and meta-analyses in the present study reported substantial variation across their included articles regarding the assessment methods (e.g., requirement of screening tools developed or validated in athlete samples; diagnoses to be confirmed via structured clinical interviews; or use of representative sampling) and the operationalisation of disordered eating (e.g., were specific disordered eating symptoms or were eating disorder diagnostic criteria assessed; were DSM-IV or DSM-5 diagnostic criteria used). These inconsistencies are particularly concerning given the general lack of consideration of risk-of-bias assessment in the interpretation of results across the included reviews, leaving space for prevalence estimates to be influenced by biased results. As such, it is difficult to synthesise precise prevalence statistics or to compare between syntheses of separate demographics (e.g., comparing syntheses for genders, age groups, and sport types) and likely contributes to the vast ranges in prevalence proportions reported (i.e., from 0% to 85.8%).

Evidently, greater consistency in assessment methods is needed for future prevalence research. At a minimum, screening tools for disordered eating, in research and in practise, should only be used if they have received validation in athlete samples. We refer to two reviews that outline such tools currently available [51,52]. Further, a screening tool was recently developed in a doctoral thesis specifically for assessing the spectrum of disordered eating in athletes, with initial validation in male and female current and former athletes [53]; however, it requires further validation and peer review. Beyond screening tools, future prevalence research should consider using representative samples, matched-control groups, and differentiation between disordered eating symptoms versus clinical diagnosis (as assessed by clinical interview). Future reviews and meta-analyses should also carefully assess and consider these factors and their impact on risk of bias in interpreting results. These changes will increase confidence in prevalence estimates in future research but also in screening of at-risk athletes in practice.

The risk factors identified across the reviews (including moderators and mediators) can guide primary, secondary, and tertiary interventions for disordered eating to reduce the associated adverse health and performance impacts in elite athletes [51]. Un-modifiable (or difficult to modify) moderating factors (e.g., gender, sport type, and career changes) can help identify which athlete groups to target for primary prevention and screening. All athletes should be considered at risk for the spectrum of disordered eating; however, certain athletes may be prioritised based on moderating factors when allocating finite resources (e.g., funding and clinical support). Additionally, changes in policy and practise that target potentially modifiable factors within an athletes’ sporting environment (e.g., pressures to lose weight) may reduce disordered eating. Several recommendations for such changes are available in the IOC guidelines for preventing REDs in athletes, with considerations at the sports organisation level (e.g., implementing rule changes that de-emphasise body shape and weight) and for health and training staff in direct contact with athletes (e.g., providing education and de-emphasising body composition [51]).

Mediating factors suggest mechanisms via which certain risk factors increase risk and can be targeted through primary prevention and tertiary intervention programs [20]. Some of the factors identified in the current review align with the established mediating factors in the general population, including internalisation of appearance ideals [54], body dissatisfaction [54], and the overvaluation of weight and shape [55]. As such, interventions for eating disorders which target these factors (e.g., CBT-E [55]; the Body Project [56]) may translate to elite athletes. Indeed, a group-based prevention program targeting internalisation of appearance ideals has shown some effectiveness in reducing body image concerns and disordered eating symptoms in female athletes [57,58], female dancers [59], and male athletes [60]. Whilst findings from these prevention programs are somewhat promising, outcomes for athletes are disappointing when compared with the general population [61,62], suggesting the presence of other factors influencing disordered eating in elite athletes. Further, evaluations of interventions for disordered eating in elite athletes is scarce, with current findings including athletes from all levels of competition and/or lacking methodological rigour or theoretical grounding [63]. Indeed, as noted in our original PROSPERO pre-registration, we initially intended to compare findings across reviews of interventions for disordered eating in elite athletes; however, interventions were only included in two reviews, with Karrer et al. (2020) [6] reporting no studies investigating treatment of disordered eating in elite male athletes and Hincapié and Cassidy (2010) [28] reporting only one non-randomised, non-controlled study in dancers. As such, the effectiveness of current interventions for the prevention or treatment of disordered eating in elite athletes remains unclear.

**Figure 2:**
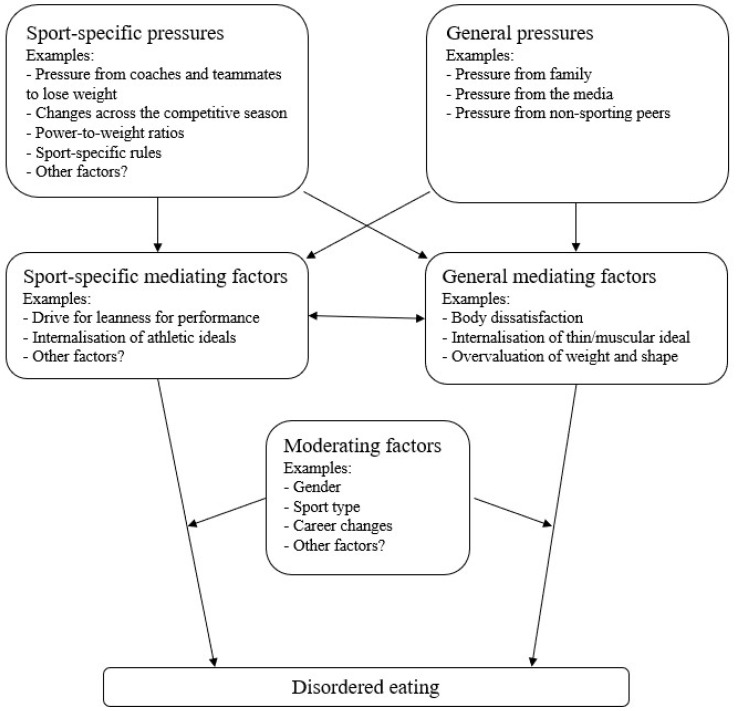
Potential hypotheses of sport-specific versus general risk factors for disordered eating in elite athletes.

Undermining this confidence further is the conspicuous research gap regarding how risk factors for disordered eating in elite athletes may converge or differ from the general population. These well-established mediating factors of internalisation of appearance ideals, body dissatisfaction, and overvaluation of weight and shape were included in only 3 of the 24 reviews [6,37,39]. Other reviews identified sport-specific pressures and mediating factors (e.g., pressure from coaches and teammates to lose weight and drive for leanness for sport performance); however, most of the sport-specific risk factors suggested by Bratland-Sanda and Sundgot-Borgen (2013) [21] were absent in the included reviews. Such factors have not yet been tested for intervention. Future longitudinal and intervention-based research is needed to investigate which sport-specific risk factors might contribute to disordered eating in elite athletes beyond those factors identified in the general population (see Figure 2). These may be informed by the various factors identified across only one review or meta-analysis (e.g., athletic identity and lack of nutritional support and education—see Table 7 for full list) or themes identified in qualitative studies [47,48,49]. Such research would inform the modification of current prevention and treatment programs to specifically target elite athlete populations.

### 4.2. Limitations

The findings from this umbrella review should be interpreted with the following considerations. Most of the included reviews and meta-analyses were at risk of bias across several critical domains. Most notably, only three reviews explicitly considered risk of bias among their included studies when interpreting findings. This undermined the confidence in the results of these reviews and clouded implications for future research and practise. Authors of future reviews should consider published guidelines for conducting and reporting systematic reviews [25]. For the current review, our research question used a narrow search criterion including only studies that reported a systematic search, were published in peer-reviewed journals, and reported on a sample of elite athletes. As such, relevant findings from non-systematic reviews or “grey literature” may have been overlooked, and it is unclear whether the findings will generalise to non-elite athletes. Additionally, some studies were included across multiple reviews, potentially biasing some of the results. However, CCA analyses indicated that this overlap was only slight.

### 4.3. Conclusions

The full spectrum of disordered eating (including eating disorders) was evident across all included reviews, and elite athletes are generally at elevated risk compared with the general population. Sports organisations should address disordered eating in policy and practise using a whole-sport approach that targets all aspects of intervention, including reducing risk factors within sporting environments (e.g., reducing a focus on weight and body shape in sport), health promotion and prevention programs, and treatment where indicated. Further research is needed to improve primary prevention and tertiary treatments targeted towards elite athletes with consideration of their unique risk factors. Secondary prevention can be achieved through implementing broad screening across the entire range of athletes; however, sporting organisations need to have adequate and appropriate clinical support systems in place to respond to and support those athletes identified through screening. Additional supports may be required for female athletes, athletes participating in lean sports, and those experiencing career changes. However, these findings should be interpreted carefully, with much of the research to date potentially limited by high risk of bias and a lack of valid and standardised assessment tools. Further research is needed to address these limitations and further clarify how risk factors may differ for elite athletes versus the general population.

## Figures and Tables

**Table 1:** Features of included studies.

Author	Objective of Review	Type of Review	Unique Participant Details or Context *	Inclusion Criteria	Number of Reviewers	Number of Databases	Date of Database Searching	Publication Date Range	Number of Studies	Instrument for Quality Appraisal
Hausenblas and Carron (1999) [14]	(1) MA of DE in athletes; (2) moderators: age, BMI, sport type, competition level	MA	Mixed competition level—separate syntheses for elite athletes	Athlete sample; comparison with control; measure of DE; data sufficient for effect size	Unclear	6; Dissertation Abstracts Online, Educational Research in Completion, MEDLINE, PsychINFO, Sociofile, SPORTDiscus	Unclear	1978–1998	92 studies	Nil reported
Smolak et al. (2000) [16]	(1) MA of eating problems in female athletes; (2) moderators: sport type, elite vs. non-elite	MA	Females; mixed competition level—separate syntheses for elite athletes	Female athletes sample compared with female non-athletes; data sufficient for MA	Unclear	2; FirstSearch and PsychLit	Unclear	1975–1996 (elite)	33 studies (19 elite samples)	Nil reported
Hausenblas and Downs (2001) [17]	(1) MA of BD in athletes; (2) moderators: gender, age, sport type, competition level, ethnicity, body composition	MA	Mixed competition level—separate syntheses for elite athletes	Athlete sample and non-athlete comparison; body image measure; data sufficient for effect size	2	5; Dissertation Abstracts Online, Educational Resources in Completion, MEDLINE, PsycINFO, Sportdiscus	Unclear	1975–2000 (unclear of elite)	78 studies (19 elite)	Nil reported
Hincapie and Cassidy (2010) [28]	Summarise the epidemiology, diagnosis, prognosis, and treatment of disordered eating, menstrual disturbances, and low bone mineral density in dancers	SR	Mostly female; dancers; mixed competition level—separate syntheses for elite dancers	Dancer sample; prevalence, correlates or intervention for DE, menstrual disturbances, or low bone density in dancers, in English, include at least 20 participants	2	8; MEDLINE, CINAHL, PsycINFO, Embase, Cochrane Central Trials Registry, MANTIS, Index to Chiropractic Literature, EBSCO	2010	1980–2010 (DE)	8 studies focused on disordered eating (2 professional, 2 pre-professional, 2 university, 2 young student)	Based on Carroll et al. (2008)
Varnes et al. (2013) [29]	(1) SR of body image in female collegiate athletes vs. non-athletes; (2) moderators: sport type, competition level	SNR	Females; collegiate athletes; USA and Canada only; mixed competition level—separate syntheses for elite athletes	Collegiate female sample and non-athlete comparison; measure of body image; peer-reviewed in English	Unclear	5; Dissertation Abstracts Online, Educational Resources in Completion, MEDLINE, PsychINFO, and SPORTDiscus	1997–July 2012	1998–2012	10 studies	Nil reported
Werner et al. (2013) [30]	(1) SR of pathogenic weight -control behaviours in young elite athletes vs. non-athletes; (2) moderators: sport type, gender	SR	Young athletes (≤25 years old)	Elite current athletes (<25 years); measure of weight concerns or weight control behaviour; peer reviewed published	2	3; PubMed, PsycINFO, Spolit	Feb 2012	1993–2011	15 studies	Nil reported
Arcelus et al. (2014) [31]	(1) Summarise the prevalence of ED in dancers; (2) moderators: methodology, dance type, change in diagnosis over time	MA	Mostly female; dancers; mixed competition (mostly elite)	Dancer sample (at least 10 participants); diagnosis or symptoms to establish caseness for ED; English journal articles	2	3; MEDLINE/PubMed, PsycINFO, Embase	Jul 2013	1985–2012 (elite)	33 studies (27 elite sample)	Nil reported
Macdougall et al. (2015) [32]	(1) Comparing para- and Olympic sport athletes for well-being	MA	Para athletes	Para-athlete sample with Olympic sport athlete comparison; peer-reviewed in English	2	8; MEDLINE, PsycINFO, Embase, AMED, CINAHL. SPORTDiscus, Scopus, and Web of Science	Sep 2014	1989–2012	12 studies (2 relating to body image)	Adapted from McMaster University guidelines and Effective Public Health Practice Project
Chapman and Woodman (2016) [33]	(1) MA of disordered eating in male athletes; (2) moderators: sport type, competitive level, measurement tool	MA	Males; mixed competition level—separate syntheses for elite athletes	Male athlete sample and non-athlete comparison; data sufficient for meta-analysis	Unclear	9; PsycINFO, PsychLit, SPORTDiscus, Science Direct, Web of Knowledge, PubMed, Ingenta-Connect, First Search, Google Scholar	1 January 2014	1986–2013 (elite)	31 studies (12 elite samples)	Nil reported
Rice et al. (2016) [34]	(1) SR of psychological wellbeing among elite-level athletes	SR	+	Adult elite athlete sample; measures of mental wellbeing; English articles	3	5; PubMed, EMBASE, SPORTDiscus, PsycINFO, Cochrane; Google Scholar	May 2015	1994–2008 (elite)	60 studies (10 relating to ED and body image)	Based on Glasziou et al. and Brown et al.
Mitchell et al. (2017) [35]	(1) MA and SR of muscle dysmorphia symptomatology in body builders; (2) investigating correlates	MA	Body builders; mixed competition level—comparisons between elite and non-elite athletes	Body builder sample with non-body builder resistance trainer comparisons; psychometrically validated measure of MD; peer-reviewed journal articles	1	8; MEDLINE, PsycINFO, CINAHL, ProQuest 5000, Scopus, PubMed, SPORTDiscus, Web of Science	Feb 2015	2002–2013 (elite)	31 studies (6 elite)	Modified scale by Downs and Black
Mainwaring and Finney (2017) [36]	(1) SR of psychological factors associated with dance injuries	SR	Dancers; mixed competition level (mostly elite)	Dancer sample; measure of injury relating to dance; measure of psychological factor related to the injury	2	4; SPORTDiscus, MEDLINE, CINAHL, PsycINFO	7 July 2014	2001–2015 (ED related)	13 studies (3 relating to DE)	Custom assessment based on Finney et al. and Lagerveld et al.
Buckley et al. (2019) [37]	(1) SR of athletes’ relationships with food and bodies after retirement from elite sport	SR	Retired elite athletes	Retired elite athlete sample; measures of eating behaviours or body image; English peer-reviewed articles	2	6; Web of Science, Scopus, PubMed, EBSCO Host, SPORTDiscus, CINAHL	Aug 2018	1996–2018	16 studies	Pluye et al. (2009)
Mancine et al. (2020) [38]	(1) SR of DE in athletes; (2) moderators: sport type	SR	Mixed levels—separate syntheses for elite athletes	Sample of athletes; standardised tool or interview for risk of DE; English peer-reviewed published work	2	1; PubMed	Jul 2019	2007–2019	12 studies (at least 7 elite)	Nil reported
Stoyel et al. (2020) [39]	(1) SR of evidence for a model of DE in athletes; (2) assess mediators for DE	SR	+	Sample of elite athletes; measure of DE; measure of a mediator from the model; quantitative peer-reviewed articles published after 2000	Unclear	4; Ovid-MEDLINE, PsycINFO, JSTOR, EBSCOhost	Nov 2018	2002–2017	37 studies	Effective Public Health Practice Project assessment tool
Karrer et al. (2020) [6]	(1) SR of DE in male elite athletes	SSR	Males	Adult elite male athlete sample; measure of DE; English or German language	2	4; PsycINFO, PubMed, SPORTDiscus, Web of Science	May 2020	Not stated	80 studies (14 controlled, 47 uncontrolled, 1 intervention, 18 reviews)	None used
King et al. (2021) [40]	(1) SR of jockeys’ mental health	SR	Professional jockeys	Professional jockey sample; data of mental health disorders or help-seeking; English language	2	2; PubMed, Google Scholar	Jan 2021	1987–2019 (DE)	16 studies (12 relating to DE)	Nil reported
Roberts et al. (2022) [41]	(1) SR of DE and EDs in competitive cyclists	SNR	Competitive cyclists; mixed competition level (majority elite)	Competitive cyclist sample; reference to ED or DE, eating patterns and attitudes, race weight, leanness; English peer-reviewed articles	2	4; PubMed, SPORTDiscus, Web of Science, Google Scholar	Sep 2022	2003–2022	14 studies	None used
Woods et al. (2022) [42]	(1) SR of prevalence of mental health symptoms among professional footballers	SNR	Professional football players	Elite footballers sample; data on mental health disorders or their symptoms; English language	3	1; MEDLINE	Unclear	2015–2021 (DE)	13 studies (5 relating to DE)	Nil reported
Chapa et al. (2022) [23]	(1) MA of ED psychopathology (i.e., DE and BIC) in female athletes; (2) moderators: sport type, age, competition level	MA	Females; mixed competition level—included as moderating variable	Female athlete sample and non-athlete comparisons; sufficient data for meta-analysis; English studies (including dissertations)	2	2; PubMed and PsycINFO	Jan 2022	2001–2021 (elite)	56 studies (18 elite)	Joanna Briggs Institute Critical Appraisal Checklist for Analytical Cross-sectional Studies
Burgon et al. (2023) [22]	(1) MA and SR of BIC in adult athletes; (2) moderators: sport type, competition level, gender	MA	Mixed competition level—separate syntheses for elite athletes	Elite adult (>16 years) athlete sample and non-athlete comparisons; quantitative measure of BIC; sufficient data for effect sizes; English quantitative study	2 (2nd checked 10%)	3; Scopus, PsycINFO; PubMed	Mar 2023	1989–2021	21 studies	Critical Appraisal Skills Programme
Godoy-Izquierdo et al. (2023) [43]	(1) SR of association between exercise addiction and DE in competitive athletes	SR	Mixed competition level—separate syntheses for elite athletes	Competitive athlete sample; measure of DE and exercise addiction; English or Spanish peer-reviewed and grey literature	4	5; Web of Science, Scopus, ProQuest, EBSCOhost (including SportDiscus and Psicodoc databases), and Cochrane Library	Apr 2020	1998–2019 (higher competition)	22 studies (10 higher competition level)	Nil reported
Zaccagni and Gualdi-Russo (2023) [44]	(1) MA and SR of BIC in athletes; (2) compare general vs. sport BIC; (3) moderators: gender, sport type, competition level; body composition	MA	Mixed competition level—comparisons of elite versus non-elite athletes	Competitive athlete sample (non-clinical sample); including body silhouette scale measure of BIC and anthropometric measurements; English journal articles	2	2; PubMed and Web of Science	2012–2022	2012–2021	15 studies	Adapted Newcastle–Ottawa Scale for observational studies
Smith et al. (2023) [45]	(1) SR of mental health of cyclists	SR	Cyclists	Cyclists; psychiatric concerns; peer-reviewed articles; in English	2	3; PsychINFO, PubMed, Scopus	March 2023	2003–2022	10 studies (8 relating to ED)	Nil reported

Note. * A “+” indicates that the sample included mixed genders, mixed ages, mixed sport types, mixed countries, and only elite athletes. Only deviations from this standard are reported in the “Unique participant details or context” column. MA = meta-analysis; SR = systematic review; SNR = systematic narrative review; SSR = systematic scoping review; DE = disordered eating; ED = eating disorder; BIC = body image concerns; MD = muscle dysmorphia.

**Table 2:** Summary of the key risk of bias concerns based on the AMSTAR 2.

Review or Meta-Analysis	Key Risk of Bias Concerns					
	Pre-Registration	Search Procedures	Justification for Excluded Studies	Risk of Bias in Interpretation of Results	Meta-Analytic Statistical Methods (Meta-Analysis)	Consideration of Publication Bias (Meta-Analysis)
Systematic Reviews without Meta-Analyses						
Hincapie and Cassidy (2010) [28]	X	X	X			
Varnes et al. (2013) [29]	X		X	X		
Werner et al. (2013) [30]	X		X	X		
Rice et al. (2016) [34]	X		X	X		
Mainwaring and Finney (2017) [36]	X		X			
Buckley et al. (2019) [37]			X	X		
Mancine et al. (2020) [38]	X	X	X	X		
Stoyel et al. (2020) [39]	X	X	X	X		
Karrer et al. (2020) [6]	X			X		
King et al. (2021) [40]	X	X	X	X		
Roberts et al. (2022) [41]	X		X	X		
Woods et al. (2022) [42]	X	X	X	X		
Godoy-Izquierdo et al. (2023) [43]	X			X		
Smith et al. (2023) [45]	X		X	X		
Systematic Reviews with Meta-Analyses						
Hausenblas and Carron (1999) [14]	X	X	X	X		
Smolak et al. (2000) [16]	X	X	X	X		X
Hausenblas and Downs (2001) [17]	X		X	X		
Arcelus et al. (2014) [31]	X			X		X
Macdougall et al. (2015) [32]	X			X	X	X
Chapman and Woodman (2016) [33]	X		X	X		
Mitchell et al. (2017) [35]	X		X	X	X	X
Chapa et al. (2022) [23]	X		X	X		
Burgon et al. (2023) [22]						
Zaccagni and Gualdi-Russo (2023) [44]		X	X	X		X

Note. An “X” indicates major risk of bias concerns in this area (e.g., not reported or unclear). Further details are available in Supplementary S2.

**Table 3:** Summary of evidence for syntheses comparing prevalence of disordered eating in elite athletes versus non-athletes.

Elevated risk of disordered eating for athletes	MA (1978–1998) of elite female athletes. DE higher in athletes than non-athletes (small effect) (Hasuenblas and Carron, 1999) [14]	MA (1975–1996) of female elite athletes. DE higher in athletes than non-athletes (small effect) (Smolak et al., 2000) [16]	* MA (1985–2012) of dancers. ED risk, including AN and EDNOS but not BN, higher in dancers than non-dancers. DE higher in dancers than non-dancers (Arcelus et al., 2014) [31]	* MA (1986–2013) of male athletes. DE higher for athletes than non-athletes for the Eating Attitudes Test (small effect) (Chapman and Woodman, 2016) [33]	* MA (1986–2013) of male athletes. DE higher for male wrestlers than non-athletes (small effect) (Chapman and Woodman, 2016) [33]
	SR (1994–2008) of elite athletes. DE and BIC higher in athletes than non-athletes (Rice et al., 2016) [34]	MA (2002–2013) of competitive body builders. MD higher in body builders than non-body builder resistance trainers (medium to large effects) (Mitchell et al., 2017) [35]	SR (2002–2017) of elite athletes. DE higher in athletes than non-athletes in 5/9 studies (no difference in 4/9) (Stoyel et al., 2020) [39]	SR (1996–2018) of retired elite athletes. DE higher in retired athletes than non-athletes (Buckley et al., 2019) [37]	SR (dates unclear) of male elite athletes. DE higher in athletes than non-athletes in 7/11 studies (Karrer et al., 2020) [6]
No significant difference in disordered eating	SR (1993–2011) of elite young (under 25 years) athletes. Mixed findings with no conclusive evidence of elevated risk of DE for athletes (Werner et al., 2013) [30]	MA (1986–2013) of male elite athletes. No difference between athletes and non-athletes for DE (Chapman and Woodman, 2016) [33]	* MA (2001–2021) of female athletes. No difference between athletes and non-athletes for DE, drive for thinness, restricting, binge eating (Chapa et al., 2022) [23]		
Lower risk of disordered eating for athletes	* MA (2001–2021) of female athletes in non-lean sports. Drive for thinness lower in athletes than non-athletes (Chapa et al., 2022) [23]				

Note. Red indicates greater risk for elite athletes versus non-athletes, grey indicates no difference in risk for elite athletes, and green indicates lower risk for elite athletes. * indicates the inclusion of both elite and non-elite athletes in the sample. SR = systematic review, MA = meta-analysis, DE = disordered eating, AN = anorexia nervosa, EDNOS = eating disorder not otherwise specified, BN = bulimia nervosa, BIC = body image concerns, MD = muscle dysmorphia.

**Table 4:** Summary of evidence for syntheses comparing body image concerns in elite athletes and non-athletes.

Lower risk of body image concerns for athletes	* MA (1975–1996) of female athletes. EDI-BD lower in athletes than non-athletes (medium effect), despite higher EDI-DT scores (small effect) (Smolak et al., 2000) [16]	MA (1975–2000) of elite athletes. BD lower in elite athlete than non-athletes (small effect) (Hausenblas and Downs, 2001) [17]	SR (1998–2012) of elite female collegiate athletes (USA). BIC lower for athletes than non-athletes (Varnes et al., 2013) [29]	SR (dates unclear) of male elite athletes. BD lower in athletes than non-athletes in 6/14 studies (no difference in 8/14) (Karrer et al., 2020) [6]
	* MA (2001–2021) of female athletes. BD lower in athletes than non-athletes (Chapa et al., 2022) [23]	* MA (2001–2021) of female athletes in non-lean sports. BD lower in athletes than non-athletes (Chapa et al., 2022) [23]	* MA (1990–2021) of athletes. BIC lower in athletes than non-athletes (medium effect) (Burgon et al., 2023) [22]	

Note. Green indicates lower risk for elite athletes. * indicates the inclusion of both elite and non-elite athletes in the sample SR = systematic review, MA = meta-analysis, EDI-BD = Eating Disorder Inventory-Body dissatisfaction, EDI-DT = Eating Disorder Inventory-Drive for thinness, BD = body dissatisfaction, BIC = body image concerns.

**Table 5:** Summary of evidence regarding risk factors for disordered eating in elite athletes across syntheses.

	General Risk Factors				Sport-Specific Factors					
	Demographics		Psychological Factors		Sport Categories	Pressures within Sport	Sport-Specific Psychological Factors	Career Changes		
	Gender	Age	BD	Negative Affect				Injuries	Performance Changes	Retirement
* MA of athletes (Hausenblas and Carron, 1999) [14]	RR vs. non-athletes: male (female)				RR vs. non-athletes: aesthetic (ball) in females					
MA of elite female athletes (Smolak et al., 2000) [16]					RR vs. non-athletes: lean (non-lean)					
SR of young elite athletes (Werner et al., 2013) [30]	Female (male)	=			Lean (non-lean)					
* MA of dancers (Arcelus et al., 2014) [31]					Ballet (other dancers)					
* MA of male athletes (Chapman and Woodman, 2016) [33]					=					
SR of elite athletes (Rice et al., 2016) [34]	Female (male)	Younger (older)			Lean (non-lean)			Risk of injury		
SR of dancers (Mainwaring and Finney, 2017) [36]								Risk of injury		
SR of retired elite athletes (Buckley et al., 2019) [37]			Self-objectification		High energy consumption		Internalisation of athletic ideals	Injury during career	Career dissatisfaction	Retirement
SR of elite athletes (Mancine et al., 2020) [38]					Lean (non-lean)					
SR of elite athletes (Stoyel et al., 2020) [39]			BD; overvaluation of weight and shape	Negative affect	Lean (non-lean)	High performance climate; Pressure from coaches and teammates	Drive for leanness in sport			
SR of elite male athletes (Karrer et al., 2020) [6]		RR vs. non-athletes: adults (younger)	BD	Depression	Lean (non-lean)				Career dissatisfaction	
SR of elite jockeys (King et al., 2021) [40]						Changes across competitive seasons				
* SR of cyclists (Robert et al., 2022) [41]					High energy consumption	Power-to-weight ratios	Internalisation of ideal cyclist body			
SR of professional footballers (Woods et al., 2022) [42]								=		Retirement
* MA of female athletes (Chapa et al., 2022) [23]		=			Lean (non-lean)					
* SR of elite athletes (exercise addiction) (Godoy-Izquierdo et al., 2023) [43]	=	=			Weight-sensitive					
SR of elite cyclists (Smith et al., 2023) [45]						Pressure from coaches and teammates; changes across competitive seasons	Drive for leanness in sport			

Note. Risk factors identified in each synthesis are presented, with reference group (if provided) in brackets. “=” indicates synthesis found no consistent evidence of a relationship between this risk factor and disordered eating. “*” indicates that this risk factor was assessed including elite and non-elite athletes in this synthesis, although there was evidence to suggest to no significant difference between elite and non-elite athletes for disordered eating. BD = body dissatisfaction, MA = meta-analysis, SR = systematic review, RR = relative risk.

**Table 6:** Summary of evidence regarding risk factors for body image concerns in elite athletes across syntheses.

	General Risk Factors			Sport-Specific Factors
	Demographics			Sport Categories
	Gender	Age	Para-Athletes	
* MA of athletes (Hausenblas and Downs, 2001) [17]	RR vs. non-athletes: =	RR vs. non-athletes: =		=
* SR of female collegiate athletes (Varnes et al., 2013) [29]				
MA of elite para-athletes (Macdougall et al., 2015) [32]			Para-athletes (non-para-athletes)	
* MA of female athletes (Chapa et al., 2022) [23]		=		Mixed findings between SR and MA
* MA of athletes (Burgon et al., 2023) [22]				Lean (non-lean) in females
* MA of athletes (Zaccagni and Gualdi-Russo, 2023) [44]	Females (males)			=

Note. Risk factors identified in each synthesis are presented, with reference group (if provided) in brackets. “=” indicates synthesis found no consistent evidence of a relationship between this risk factor and disordered eating. “*” indicates that this risk factor was assessed including elite and non-elite athletes in this synthesis, although there was evidence to suggest to no significant difference between elite and non-elite athletes for disordered eating. MA = meta-analysis, SR = systematic review, RR = relative risk.

**Table 7:** Potential risk factors for disordered eating which were only included in one review or meta-analysis.

Article	Risk Factor
SR of elite athletes (Rice et al., 2016) [34]	Commencement of training at an earlier age
SR of retired elite athletes (Buckley et al., 2019) [37]	Athletic identity
SR of retired elite athletes (Buckley et al., 2019) [37]	Comparison with other athletes
SR of retired elite athletes (Buckley et al., 2019) [37]	Involvement in sport following retirement
SR of dancers (Hincapie & Cassidy, 2010) [28]	Body fat outside of “normative” range for dancers
SR of dancers (Hincapie & Cassidy, 2010) [28]	Lower bone mineral density
SR of dancers (Hincapie & Cassidy, 2010) [28]	Male-typified gender role
SR of dancers (Hincapie & Cassidy, 2010) [28]	Living alone
SR of cyclists (Roberts et al., 2022) [41]	Lack of nutritional support
SR of elite male athletes (Karrer et al., 2020) [6]	Other-oriented perfectionism
SR of elite athletes (Stoyel et al., 2020) [39]	Internalisation of appearance ideals

## Data Availability

No new data were created or analysed in this study. Data sharing is not applicable to this article.

## Supplementary Materials

The following supporting information can be downloaded at: https://www.mdpi.com/article/10.3390/jcm13144171/s1. Supplementary S1: Full search strategy; Supplementary S2: Risk of bias assessment of the included studies using the AMSTAR 2 [6,14,16,17,22,23,28,29,30,31,32,33,34,35,36,37,38,39,40,41,42,43,44,45].
