# Supplementary material for: An Umbrella Review of Body Image Concerns, Disordered Eating, and Eating Disorders in Elite Athletes
Source: J Clin Med. 2024 Jul 16;13(14):4171. doi: 10.3390/jcm13144171 (PMC11278087; doi:10.3390/jcm13144171)
Supplement: Supplementary file 1 [file jcm-13-04171-s001.zip › jcm-3066724-supplementary.pdf]

Supplementary S1: Full search strategy

Following consultation with a health librarian, searches were originally completed on four electronic databases: CINAHL (EBSCO), PsychINFO (OVID), MEDLINE (OVID), and Scopus, including all years until the search date, July 4th, 2023. An updated search of these databases and the addition of an extra database, SPORTDiscus (EBSCO), was conducted on January 9th, 2024. A search strategy was used following the PCC method.

Population was searched using relevant index terms (Athletes/) OR searching the title and abstract for (athlete* OR olympian OR player* OR sport*).

Concept was searched using relevant index terms (Eating Disorders/ OR “Feeding and Eating Disorders”/ OR relative energy deficiency in sport/) OR searching title and abstract for ("eating disorder" OR "disordered eating" OR "body image" OR "body dissatisfaction" OR dietary restrict* OR calorie restrict* OR purg* OR binge* OR "muscle dysmorphi*" OR anorexi* OR bulimi* OR "OSFED" OR "UFED" OR "EDNOS" OR "night eating" OR "relative energy deficiency in sport").

Context was searched using relevant index terms (systematic review/ OR meta-analysis/) OR searching title and abstract for (review OR meta*).

Searches were then limited to English peer-reviewed articles.

Supplementary S2. Risk of bias assessment of the included studies using the AMSTAR 2.

	AMSTAR 2 item number															
	1	2	3	4	5	6	7	8	9	10	11	12	13	14	15	16
Hausenblas & Carron (1999) [14]	Yes	No	Yes	No	No	No	No	Yes	No	No	Yes	No	No	Yes	Yes	No
Smolak et al. (2000) [16]	Yes	No	No	No	No	No	No	Yes	No	No	Yes	No	No	Yes	No	No
Hausenblas & Downs (2001) [17]	Yes	No	Yes	Partial-yes	No	Yes	No	No	No	No	Yes	No	No	Yes	Yes	No
Hincapie & Cassidy (2010) [28]	Yes	No	Yes	No	Yes	No	No	Yes	Yes	No	N/A	N/A	Yes	No	N/A	Yes
Varnes et al. (2013) [29]	Yes	No	Yes	Partial-yes	No	No	Partial-yes	Yes	No	No	N/A	N/A	No	Yes	N/A	No
Werner et al. (2013) [30]	Yes	No	No	Partial-yes	Yes	No	No	Yes	No	No	N/A	N/A	No	Yes	N/A	Yes
Arcelus et al. (2014) [31]	Yes	No	Yes	Partial-yes	Yes	Yes	Partial-yes	Yes	No	No	Yes	Yes	No	No	No	No
Macdougall et al. (2015) [32]	Yes	No	Yes	Partial-yes	Yes	Yes	Partial-yes	Yes	Yes	No	No	No	No	Partial-yes	No	No
Chapman & Woodman (2016) [33]	Yes	No	Yes	Partial-yes	No	No	No	Yes	No	No	Yes	No	No	Yes	Yes	Yes
Rice et al. (2016) [34]	Yes	No	No	Partial-yes	Yes	No	No	Partial-yes	Yes	No	N/A	N/A	No	Yes	N/A	Yes
Mitchell et al. (2017) [35]	Yes	No	Yes	Partial-yes	No	Yes	Partial-yes	Yes	Yes	No	No	No	No	Yes	No	Yes
Mainwaring & Finney (2017) [36]	Yes	No	No	Partial-yes	Yes	No	No	Yes	Yes	No	N/A	N/A	Yes	Yes	N/A	No

Buckley et al. (2019) [37]	Yes	Yes	Yes	Partial-yes	Yes	No	No	Yes	Yes	No	N/A	N/A	No	No	N/A	Yes
Mancine et al. (2020) [38]	Yes	No	No	No	Yes	No	No	Partial-yes	No	No	N/A	N/A	No	No	N/A	Yes
Stoyel et al. (2020) [39]	Yes	No	No	No	No	No	No	Yes	Yes	No	N/A	N/A	No	Yes	N/A	No
Karrer et al. (2020) [6]	Yes	No	Yes	Partial-yes	Yes	Yes	Partial-yes	Partial-yes	No	No	N/A	N/A	No	Yes	N/A	Yes
King et al. (2021) [40]	Yes	No	No	No	Yes	No	No	Yes	No	No	N/A	N/A	No	No	N/A	Yes
Roberts et al. (2022) [41]	Yes	No	No	Partial-yes	Yes	Yes	No	Yes	No	No	N/A	N/A	No	No	N/A	Yes
Woods et al. (2022) [42]	Yes	No	No	No	Yes	No	No	Yes	No	No	N/A	N/A	No	No	N/A	No
Chapa et al. (2022) [23]	Yes	No	Yes	Partial-yes	Yes	Yes	No	Yes	Yes	No	Yes	No	No	Yes	Yes	No
Burgon et al. (2023) [22]	Yes	Yes	Yes	Partial-yes	Yes	Yes	Partial-yes	Yes	Yes	No	Yes	Yes	Yes	Yes	Yes	Yes
Godoy-Izquierdo et al. (2023) [43]	Yes	No	Yes	Partial-yes	Yes	Yes	Partial-yes	Yes	No	No	N/A	N/A	No	Yes	N/A	Yes
Zaccagni & Gualdi-Russo (2023) [44]	Yes	Yes	No	No	Yes	No	Partial-yes	Yes	Yes	No	Yes	No	No	Yes	No	Yes
Smith et al. (2023) [45]	Yes	No	No	Partial-yes	No	Yes	No	Yes	No	No	N/A	N/A	No	No	N/A	Yes
